# The interconnected wealth of nations: Shock propagation on global trade-investment multiplex networks

**DOI:** 10.1038/s41598-019-49173-2

**Published:** 2019-09-11

**Authors:** Michele Starnini, Marián Boguñá, M. Ángeles Serrano

**Affiliations:** 1 ISI Foundation, Torino, Italy; 2 Departament de Física de la Matèria Condensada, Universitat de Barcelona, Martí i Franquès 1, 08028 Barcelona, Spain; 3 Universitat de Barcelona Institute of Complex Systems (UBICS), Universitat de Barcelona, Barcelona, Spain; 4 ICREA, Pg. Lluís Companys 23, E-08010 Barcelona, Spain

**Keywords:** Computational science, Complex networks

## Abstract

The increasing integration of world economies, which organize in complex multilayer networks of interactions, is one of the critical factors for the global propagation of economic crises. We adopt the network science approach to quantify shock propagation on the global trade-investment multiplex network. To this aim, we propose a model that couples a spreading dynamics, describing how economic distress propagates between connected countries, with an internal contagion mechanism, describing the spreading of such economic distress within a given country. At the local level, we find that the interplay between trade and financial interactions influences the vulnerabilities of countries to shocks. At the large scale, we find a simple linear relation between the relative magnitude of a shock in a country and its global impact on the whole economic system, albeit the strength of internal contagion is country-dependent and the inter-country propagation dynamics is non-linear. Interestingly, this systemic impact can be associated to intra-layer and inter-layer scale factors that we name network multipliers, that are independent of the magnitude of the initial shock. Our model sets-up a quantitative framework to stress-test the robustness of individual countries and of the world economy.

## Introduction

The integrated nature of the world economy is the ultimate cause for the propagation of economic crisis at a global scale^1^. A shock originated in a country may spread to its economic partners through multiple channels, captured by the balance of payments^2,3^. Shocks can have different origins, e.g. financial shocks can be caused by defaults of big financial institutions or sovereign debt crisis, while trade shocks may be triggered by barriers raised by governments, such as protective tariffs. The increasing global interconnectedness of world economies^4^ calls for a modeling framework of shock propagation able to incorporate the full complexity of these interactions^5–7^.

Network science^8^ provides useful tools to quantify, model, and predict the behavior of spreading phenomena in complex systems, from information diffusion over social networks to epidemics in living systems^9,10^. The study of international trade networks, in particular, has a long tradition in network science^11–18^. Network effects have been showed to substantially affect the spreading of economic crises^19–21^, while network tools are being increasingly employed to estimate systemic risk among financial institutions^22–28^ and to evaluate financial contagion over networks of banks^29–32^. Most of these works assess financial stability by considering the failure of single institutions (e.g., banks) and specific propagation channels (e.g. interbank lending), and only a few works considered global networks at the country level^33,34^ and addressed shock propagation over financial cross-border networks^35–37^.

Since international shocks spread through both trade and financial channels, neglecting the interplay between them may lead to underestimate spillover effects. In this paper, we address global shock propagation by taking into account both trade and financial international relations, represented as a multiplex network^38^. The trade-investment multiplex is reconstructed using yearly data of bilateral trade and financial positions between countries, coupled to a dynamics describing how economic distress is transmitted from one country to another. Our model allows us to estimate both the vulnerability of a country to external shocks, and the systemic impact that a country poses to the whole economic system. Remarkably, we find that spillover effects due to the interconnectedness of trade and financial economic relations can be encoded into networks multipliers.

## The Trade-Investment Multiplex Network

Trade and investment interactions between countries in the world can be represented in two different layers of a multiplex network, the global trade-investment (GTI) network. Multi-layered networks provide a proper theoretical framework to address contagion phenomena involving different propagation channels, whose interplay cannot be captured by single-layered networks^39,40^. In the GTI multiplex network, countries are represented as nodes and weighted links represent the existence and intensity of trade or financial connections between countries, and are separated in two different layers, one for each type of economic interaction. In the trade (*T*) layer, the amount of goods exported from country *i* to country *j* can be taken as a proxy for the bilateral vulnerability of *i* with respect to a shock originated in *j*: the more country *i* exports to country *j*, the more country *i* will be affected by a demand drop in country *j*. We reconstruct the *T* layer by means of bilateral trade data of goods exchanges, by using the United Nations Commodity Trade Statistics Database^41^ (analyzed for the first time as a complex network in^11^), as detailed in the Methods section. A directed link from country *i* to country *j* in layer *T* represents the exports of goods from *i* to *j*, *x*_*ij*_, in a given year, which is equivalent to the imports of goods of *j* from *i*, ${m}_{ji}\equiv {x}_{ij}$. The total exports of country *i* are simply ${X}_{i}={\sum }_{j}\,{x}_{ij}$, and its total imports are ${M}_{i}={\sum }_{j}\,{x}_{ji}={\sum }_{j}\,{m}_{ij}$. Transactions in goods account for the majority (generally over 70%) of the current account of a country, and thus they can be considered as a good proxy of the strength of its trading interactions, see Supplementary Material (SM) for details.

In the investment (*I*) layer, the bilateral vulnerability of a country *i* with respect to a shock originated in a country *j* can be accounted for by the financial dependence of *i* on *j*: the more *i* relies on *j*’s investments to finance its economy, the more country *i* will be directly affected by a shock originating in country *j*. Here, we consider cross-border positions of portfolio securities between two countries, reported in ref. ^42^, as a proxy of the strength of their financial bilateral exposure, see Methods and SM. The *I* layer is thus reconstructed such that a link directed from node *i* to node *j* represents the stock of portfolio assets owned by country *i* and issued by country *j* in a given year, *a*_*ij*_, equivalent to a portfolio liability for *j* to *i*, ${l}_{ji}\equiv {a}_{ij}$. The total stock of portfolio assets owned by *i* in a given year is simply ${A}_{i}={\sum }_{j}\,{a}_{ij}$, and its total portfolio liabilities reads ${L}_{i}={\sum }_{j}\,{a}_{ji}={\sum }_{j}\,{l}_{ij}$. Note that while the *T* layer is formed by trade flows, links in the *I* layer represent stock quantities. Finally, note that the following trivial relations hold

1 $${W}_{T}=\sum _{i}\,{X}_{i}=\sum _{i}\,{M}_{i},\,{W}_{I}=\sum _{i}\,{A}_{i}=\sum _{i}\,{L}_{i},$$

where *W*_*T*_ stands for the annual total value of traded goods, and *W*_*I*_ for the annual total value of investment positions.

We reconstructed GTI multiplex networks for each year between 2001 and 2008. Notice that bilateral data disclosing international financial exposures are scarce and affected by important biases, like those introduced by the effects of offshore tax heavens, which could greatly affect the reliability of the results. Therefore, we decided to consider the high-quality data sets available in^42^ (stopping in 2008 right before the last crisis), which completed financial data collected by the International Monetary Fund (IMF), see Methods and SM. In the rest of the paper, we consider the GTI network corresponding to the year 2005 (whose topological properties are described in SM) as an illustrative case, results for other years are similar.

## The Shock Propagation Model

A shock in an epicenter country may be driven by different domestic or exogenous factors, such as political instability, fiscal contraction, banking crisis, etc. Here, we assume that shocks cause a drop on aggregate demand^43^. This implies that the epicenter country may reduce its imports from other countries and/or its investment in financial assets issued by other countries. An initial shock in a country *i* can be fully characterized by two parameters *α* and *β*, representing the initial variations in imports and foreign assets investment, $\delta {M}_{i}=\alpha$ and $\delta {A}_{i}=\beta$, respectively. The notation *δY*_*i*_ stands for the relative variation of the quantity *Y*_*i*_, where ${Y}_{i}=\{{X}_{i},{M}_{i},{A}_{i},{L}_{i}\}$. With this notation, an epicenter country *i* that reduces its import by 10% and investment by 20% is characterized by $\alpha =-\,0.1$ and $\beta =-\,0.2$. The distress is subsequently distributed from country *i* to its partners, proportionally to the intensities of the corresponding economic interactions. This implies negative variations in the exports and liabilities –*δX*_*j*_ and *δL*_*j*_– of impacted neighbors *j*. On their turn, these variations may produce variations in imports, *δM*_*j*_, and assets acquisition, *δA*_*j*_, of each country *j*, that will be again distributed proportionally to their neighbors in the GTI multiplex network, and so on. Therefore, the model’s behavior is defined by a coexistence of two coupled but different dynamics: i) the external propagation of the shock from distressed to connected countries, and ii) the internal contagion of the shock within distressed countries.

The inter-country contagion runs on top of GTI multiplex network and accounts properly for reverberation and second-order effects. Akin to general spreading models in network science^10^, each country is classified in three mutually exclusive states: vulnerable to receive the shock for the first time, active if it has accumulated distress and is able to propagate it, or inactive when it can receive distress from its partners but cannot propagate it anymore. Initially, all countries are in the vulnerable state, except for the epicenter country, which is active. Active nodes spread their distress to all neighbors (regardless of their status) as described in the previous paragraph, and turn inactive immediately after. Vulnerable countries reached by the propagation become active. The shock propagation continues until all active countries have spread their accumulated distress, and the active state disappears from the system. Then, the contagion dynamics is repeated several times, each time setting as initial variations the distress accumulated by inactive nodes in the last round, until the system reaches a final steady state (see SM for a concrete example). Therefore, each step of the contagion corresponds to a time interval obtained by dividing the total duration of the shock propagation dynamics, assumed to be one year, by the number of contagion steps, $n=50$, that ensures that the system has reached a steady state. See SM and Fig. S3 for details.

The internal contagion of the shock within distressed countries can be modeled by relating the variation of imports and assets acquisition of a country to the variation in its exports and liabilities incurrence in the short term, such as $\delta {M}_{t}=f(\delta {X}_{i},\delta {L}_{i})$ and $\delta {A}_{t}=f(\delta {X}_{i},\delta {L}_{i})$. By considering balance of payments constraints, indeed, the capacity of a country to pay for its import and to acquire foreign assets may depend on its ability to generate sufficient revenues from exports and financial liabilities. We thus assume that a country’s revenues from exports and financial liabilities can be viewed as a budget constraint on its capacity to import and acquire foreign assets^44^. We also assume that the exchange rate and prices do not adjust quickly and we neglect possible policy action aimed at counteract the shock effects.

Therefore, the dependency of imports and financial assets on revenues from exports and liabilities can be viewed as a simple elasticity relation, and learned from the data. To this aim, for each country we consider a multivariate linear regression model representing the correlations between the quantities *δX*_*t*_, *δM*_*t*_, *δA*_*t*_, and *δL*_*t*_. The elasticity relations can be described by the following equations (we omit the dependency in the country *i* for brevity):

2 $$\begin{array}{rcl}\delta M & = & {c}_{M}+{c}_{MX}\,\delta X+{c}_{ML}\,\delta L+{\varepsilon }_{M}\\ \delta A & = & {c}_{A}+{c}_{AX}\,\delta X+{c}_{AL}\,\delta L+{\varepsilon }_{A},\end{array}$$

where *c*_*M*_ and *c*_*A*_ represent the intercept terms and the coefficients *c*_*MX*_, *c*_*AL*_, *c*_*ML*_, and *c*_*AX*_ encode the correlations between (*δX*, *δL*) and (*δM*, *δA*), while *ε*_*M*_ and *ε*_*A*_ account for Gaussian noise, with zero average $\langle {\varepsilon }_{M}\rangle =\langle {\varepsilon }_{A}\rangle =0$ and variance $\langle {\varepsilon }_{M}^{2}\rangle ={\sigma }_{{\varepsilon }_{M}}^{2}$, $\langle {\varepsilon }_{A}^{2}\rangle ={\sigma }_{{\varepsilon }_{A}}^{2}$. It is worth to note that Eq. (2) incorporates the mechanical accounting constraint linking trade deficit/surplus with variations in part of the financial account (portfolio investments). Coefficients *c*_*MX*_, *c*_*ML*_, *c*_*AX*_, and *c*_*AL*_ thus play the role of *internal pass-through coefficients*, since they describe how the variations of imports and asset of a country depend on the variations of its exports and liabilities. It is important to remark that (i) Eqs. (2) are treated as simultaneous equations, by incorporating the possible correlations between all variables, (ii) the statistical significance of pass-through coefficients is implicitly encoded in the variances of the noise (${\sigma }_{{\varepsilon }_{M}}^{2}$ and ${\sigma }_{{\varepsilon }_{A}}^{2}$), the larger the noise, the less significant the associated coefficient. See Methods and SM for further details. Countries with internal pass-through coefficients smaller/larger than one will reduce/increase the impact of the shock to their commercial or financial partners, acting thus as absorbers/amplifiers. For instance, oil exporters play the role of shock blocker, having small internal pass-through coefficients. See Methods and SM for a detailed description of internal pass-through coefficients and their estimation.

## Systemic Vulnerability of Countries to Propagating Shocks

The shock propagation model allows us to assess the impact of demand shocks in one or more countries on the rest of the world, when the shock spreads from one country to another through international macroeconomics networks like the GTI multiplex. The impact on a country *i* produced by a shock originated in an epicenter country *E*, with parameters (*α*,*β*), can be quantified by considering the relative variations $\Delta {Y}_{i}(\alpha ,\beta ,E)$ of each macroeconomic variable of country *i*, ${Y}_{i}=\{{X}_{i},{M}_{i},{A}_{i},{L}_{i}\}$, measured at the end of the system’s evolution (once the shock has been totally absorbed by the entire system) ${Y}_{i}^{F}$, with respect to its initial value ${Y}_{i}^{I}$, that is,

3 $${V}_{i}(Y|\alpha ,\beta ,E)\equiv \Delta {Y}_{i}(\alpha ,\beta ,E)=\frac{{Y}_{i}^{F}-{Y}_{i}^{I}}{{Y}_{i}^{I}}.$$

The quantity ${V}_{i}(Y|\alpha ,\beta ,E)$ gives a measure of the vulnerability of country *i* to a shock originated in country *E*. This magnitude can be very heterogeneous across different countries and, even for the nearest neighbors of the epicenter country *E*, it incorporates systemic effects beyond direct bilateral economic interactions. By running several numerical simulations of the model with the same initial conditions (*α*, *β*, *E*), one can obtain probability distributions for the quantities ${V}_{i}(Y|\alpha ,\beta ,E)$, and consequently the average 〈*V*_*i*_(*Y*|*α*, *β*, *E*)〉 and value at risk $VaR[{V}_{i}(Y|\alpha ,\beta ,E)]$, as measures of the expected variability and the risk of loss.

**Figure 1:**
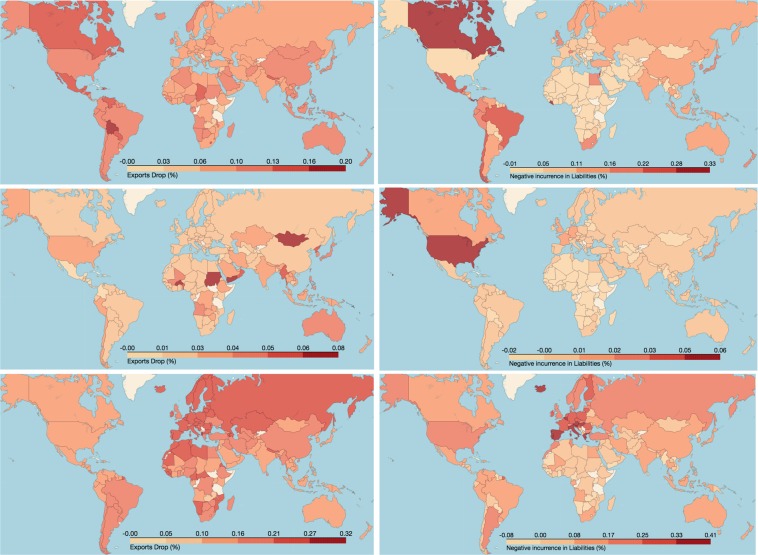
Systemic vulnerability of countries with respect to a shock originated in the United States (first row), China (second row), and in countries belonging to the EZ (third row). We used $\alpha =-\,0.4$ and $\beta =-\,0.1$. Colors indicate the VaR of exports, $VaR[\Delta {X}_{i}]$ (left plots), and of incurrence in liabilities, $VaR[\Delta {L}_{i}]$ (right plots).

Figure 1 shows the heterogeneity of the vulnerability ${V}_{i}(Y|\alpha ,\beta ,E)$ across the world, for a shock characterized by parameters $(\alpha =-\,0.1,\beta =-\,0.4)$ (different values are tested in the rest of the paper and the SM) and three different epicenter countries: the United States, China, and the Eurozone (EZ). We plot both the impacts on trade and investment, by coloring countries according to their VaR of exports, $VaR[{V}_{i}(X|-\,0.1,-\,0.4,E)]$ (left plots), and incurrence in liabilities, $VaR[{V}_{i}(L|-\,0.1,-\,0.4,E)]$ (right plots). One can see that American countries are more vulnerable to a shock originated in the United States (first row), with respect to both trade (the exports of Mexico, Canada and other South American countries may drop more than 25%) and investment. Shocks in China (second row) have a considerable lower impact on the rest of the world, especially with respect to investments. Australia, some African and South American countries may be forced to reduce their exports (probably raw materials) up to 20%, while the United States shows one of the largest reduction of foreign investment, around 10%. The economic impact on trade of a shock involving all Eurozone countries (third row) is homogeneously distributed to the rest of the world, with a general reduction of exports around 30%, while the financial impact is much more heterogeneous: most vulnerable countries are Southern European countries, forced to reduce their liabilities by more than 40%, probably due to sovereign debt exposures. The average vulnerabilities $\langle {V}_{i}(X|-\,0.1,-\,0.4,E)\rangle$ and $\langle {V}_{i}(L|-\,0.1,-\,0.4,E)\rangle$ show qualitatively similar behaviors, see SM. The model can thus be used to rank the vulnerabilities of different countries with respect to economic shocks, depending on its epicenter and magnitude.

## Quantifying Systemic Impact of Epicenter Countries

Beyond country vulnerabilities, our model allows us to measure the potential risk that each country poses for the international economic system as a whole. The global impact of a shock in a given country across the GTI multiplex can be quantified by defining its *systemic impact* ${{\mathcal{S}}}_{i}(\alpha ,\beta )$, as the total economic value that is affected by a shock originated in country *i* with parameters (*α*, *β*). The systemic impact is expected to depend crucially on the propagation of the shock from financial to trade layer, and vice versa. These spillover effects between layers can be addressed by considering separately the impacts on trade and investment. One can define the systemic impact on trade, ${{\mathcal{S}}}_{i}^{T}(\alpha ,\beta )$, and investment ${{\mathcal{S}}}_{i}^{I}(\alpha ,\beta )$, as the affected value of traded goods and financial securities expressed as a fraction of the global value of traded goods *W*_*T*_ and financial securities *W*_*I*_, respectively, that is

4 $$\begin{array}{lllll}{{\mathcal{S}}}_{i}^{T}(\alpha ,\beta ) & = & \frac{{\sum }_{j}\,\langle \Delta {X}_{j}(\alpha ,\beta ,i)\rangle }{{W}_{T}} & = & \frac{{\sum }_{j}\,\langle \Delta {M}_{j}(\alpha ,\beta ,i\rangle }{{W}_{T}},\\ {{\mathcal{S}}}_{i}^{I}(\alpha ,\beta ) & = & \frac{{\sum }_{j}\,\langle \Delta {L}_{j}(\alpha ,\beta ,i)\rangle }{{W}_{I}} & = & \frac{{\sum }_{j}\,\langle \Delta {A}_{j}(\alpha ,\beta ,i)\rangle }{{W}_{I}}.\end{array}$$

Note that the second equality holds because of Eq. (1).

**Figure 2:**
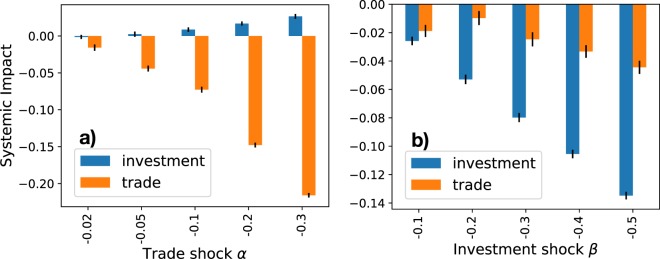
Systemic impact on trade, ${{\mathcal{S}}}_{i}^{T}(\alpha ,\beta )$, and investment, ${{\mathcal{S}}}_{i}^{I}(\alpha ,\beta )$, of a shock originated in the United States. Different combinations of values (*α*,*β*) are considered: the initial shock can be originated in the trade layer (plot (**a**)), *α* = 0, $\beta  < 0$), or trade layer (plot (**b**)), *β* = 0, $\alpha  < 0$). Error bars represent the standard error of the mean over 100 runs. A financial shock reducing by 40% the foreign assets demand in a single, large country such as the United States is expected to reduce the total value of financial securities by 11%, but also the total traded goods by 4%.

Figure 2 shows the systemic impact on trade, ${{\mathcal{S}}}_{i}^{T}(\alpha ,\beta )$, and investment, ${{\mathcal{S}}}_{i}^{I}(\alpha ,\beta )$, of a shock originated only in the financial layer (*α* = 0, Fig. 2a), or trade layer (*β* = 0, Fig. 2b) of the GTI multiplex networks, with the United States as epicenter country. As expected, the larger the initial distress, represented by parameters (*α*, *β*), the larger the systemic impact on the rest of the world. Even if the initial shock only involves one layer, the economic distress spreads from the financial to the trade layer, and viceversa. Interestingly, the different magnitudes of systemic impact reported in Fig. 2 can be quantitatively explained by our model, as we will see in the next section.

**Figure 3:**
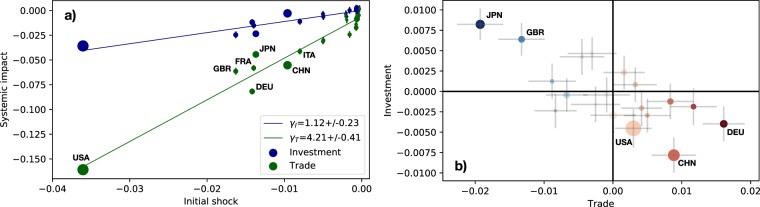
(**a**) Systemic impact on global trade ${{\mathcal{S}}}_{i}^{T}$ and investment ${{\mathcal{S}}}_{i}^{I}$, as a function of the magnitude of the initial shock ${{\mathcal{I}}}_{i}=(\alpha {M}_{i}+\beta {A}_{i})/({W}_{T}+{W}_{I})$. (**b**) Trade (${{\mathcal{D}}}_{i}^{T}$, x-axis) versus financial (${{\mathcal{D}}}_{i}^{I}$, y-axis) deviations, as obtained by plot (**a**)). The initial shock is characterized by $\alpha =\beta =-\,0.2$ (different values in the SM), countries belonging to the *G*_20_ group are shown. Error bars represent the standard error of the mean for ${{\mathcal{S}}}_{i}$. Regression coefficients ${\gamma }_{\ell }$ are plotted with 95% CI. Size of dots is proportional to countries’ GDP.

Different countries exhibit different magnitudes of systemic impact on trade or investment, that can be taken as a measure of their relevance for the stability of the GTI multiplex network. The systemic impact of a country *i*, indeed, is expected to depend on the economic value of the initial shock ${\mathcal{I}}_{i}$, determined simply as ${{\mathcal{I}}}_{i}=(\alpha {M}_{i}+\beta {A}_{i})/({W}_{T}+{W}_{I})$. Figure 3a shows the systemic impact on trade ${{\mathcal{S}}}_{i}^{T}$, and investment ${{\mathcal{S}}}_{i}^{I}$, as a function of the value of the initial shock ${\mathcal{I}}_{i}$, characterized by $\alpha =\beta =-\,0.2$, for countries belonging to the *G*_20_ group. Surprisingly, we found that the systemic impacts of these countries on global trade ($\ell =T$) or investment ($\ell =I$) are well fitted by linear regressions, whose coefficients ${\gamma }_{\ell }(\alpha ,\beta )$ represent scale factors for the initial shock. This implies that, at least for big economies, the systemic impact of a country *i* can be described simply as ${{\mathcal{S}}}_{i}^{\ell }(\alpha ,\beta )\simeq {\gamma }_{\ell }(\alpha ,\beta ){\mathcal{I}}_{i}$, where ${\gamma }_{\ell }(\alpha ,\beta )$ encodes the sensitivity of the GTI network to the shock. The larger the coefficients ${\gamma }_{\ell }(\alpha ,\beta )$, the larger the shock amplification. Notice that these coefficients depend on the initial shock but are country-independent. Even if the elasticity relations (2), determining the internal contagion within countries, are linear, the pass-through coefficients are quite heterogeneous across countries (see SM), and the inter-country propagation phase modeled by the spreading dynamics introduces highly non-linear effects.

Furthermore, it is interesting to consider the regression residuals of different countries. For each country *i*, one can define the deviations of the systemic impact of each country from the expected value obtained by the fitting function, as

5 $${{\mathcal{D}}}_{i}^{\ell }(\alpha ,\beta )={\gamma }_{\ell }(\alpha ,\beta ){\mathcal{I}}_{i}-{{\mathcal{S}}}_{i}^{\ell }(\alpha ,\beta ).$$

The trade (financial) deviation ${{\mathcal{D}}}_{i}^{T}$ (${{\mathcal{D}}}_{i}^{I}$) of a country *i* can be positive, if its systemic impact on trade (investment) is smaller than the fitted value, or negative, if ${{\mathcal{S}}}_{i}^{T}$ (${{\mathcal{S}}}_{i}^{I}$) is larger than what expected by considering the magnitude of the initial shock ${\mathcal{I}}_{i}$. Figure 3b shows the trade and financial deviations, ${{\mathcal{D}}}_{i}^{T}$ and ${{\mathcal{D}}}_{i}^{I}$, respectively, of the systemic impact of each country *i* belonging to the *G*_20_ group. These deviations are affected by both the statistical error on the systemic impact and the uncertainty of the fitting function, and thus few countries show statistically significant values of ${D}_{i}^{\ell }(\alpha ,\beta )$. However, one can see that countries having a significant, positive deviation on trade, generally show a significant, negative deviation on investment, and viceversa. China and Germany, for instance, have a larger systemic impact on trade and a smaller impact on investment than expected, while the United Kingdom and Japan show a considerably smaller impact on trade and a larger impact on investment. Even though ${D}_{i}^{\ell }(\alpha ,\beta )$ are expected to depend on the magnitude of the initial shock, these countries presenting significant values of the deviations have qualitatively similar behavior regardless the value of (*α*, *β*), as shown in the SM. It is worth to note that it is not possible to verify the linear scaling between initial shock and systemic impact, and consequently its deviations, for small economies, due to large uncertainties over the impact of these countries.

## Network Multipliers Encode Systemic Impact

**Figure 4:**
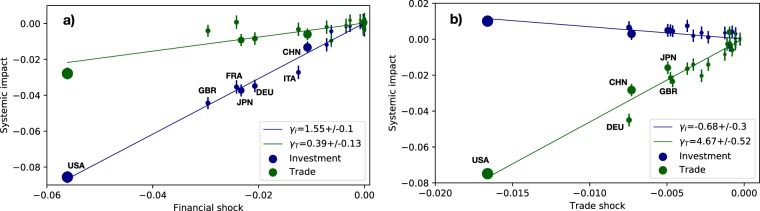
Systemic impact on global trade ${{\mathcal{S}}}_{i}^{T}$ and investment ${{\mathcal{S}}}_{i}^{I}$, as a function of the an initial shock ${\mathcal{I}}_{i}^{\ell }/{W}_{\ell }$ originated only in the investment (plot (**a**)), *α* = 0, *β* = −0.3) or trade (plot (**b**)), *α* = −0.1, *β* = 0, right) layer, for countries belonging to the *G*_20_ group. Different values of (*α*, *β*) are shown in the SM. Error bars represent the standard error of the mean for ${{\mathcal{S}}}_{i}$. Regression coefficients ${\gamma }_{\ell ^{\prime} \to \ell }$ are plotted with 95% CI. Size of dots is proportional to countries’ GDP.

The value of the coefficients ${\gamma }_{\ell }(\alpha ,\beta )$ depends on the parameters (*α*, *β*) characterizing the initial shock (see SM). One can understand this dependency by considering separately shocks originated only in one layer, investment or trade, of the GTI multiplex. Figure 4 shows that, also in the case of a exclusively financial ($\alpha =-\,0.1$, Fig. 4a) or exclusively trade ($\beta =-\,0.3$, Fig. 4b) shock, the systemic impacts ${{\mathcal{S}}}_{i}^{T}$ and ${{\mathcal{S}}}_{i}^{I}$ are well fitted by linear regressions. However, the regression coefficients do not strongly depend on the magnitude of the initial shock, being remarkably similar for different values of (*α*, *β*), see SM. Therefore, we name the scale factors ${\gamma }_{\ell ^{\prime} \to \ell }$ as *intra- and inter-layer network multipliers*, as they gauge the network effects of shock propagation from layer $\ell$ to layer $\ell ^{\prime}$ on GTI networks,

6 $${{\mathcal{S}}}_{i}^{\ell }(\alpha ,\beta )\simeq {\gamma }_{\ell ^{\prime} \to \ell }\,{\mathcal{I}}_{i}^{\ell ^{\prime} },$$

where $${\mathcal{I}}_{i}^{T}=\alpha {M}_{i}/{W}_{T}$$ and $${\mathcal{I}}_{i}^{I}=\beta {A}_{i}/{W}_{I}$$.

Figures 4, S9 and S10 show that a financial shock has an *intra-layer* network multiplier ${\gamma }_{I\to I}\simeq 1.5\pm 0.1$, and a *inter-layer* network multiplier ${\gamma }_{I\to T}\simeq 0.3\pm 0.15$. As expected, the network multiplier for the systemic impact on the investment layer, ${\gamma }_{I\to I}$, is much larger than the one for the trade layer, ${\gamma }_{I\to T}$. Conversely, a trade shock shows an intra-layer network multiplier of ${\gamma }_{T\to T}\simeq 4.5\pm 0.5$, and an inter-layer network multiplier of ${\gamma }_{T\to I}\simeq -\,0.6\pm 0.3$. It is interesting to note that the network multiplier giving the systemic impact on trade for a trade shock ${\gamma }_{T\to T}$ is much bigger than the network multiplier giving the systemic impact on investment for an investment shock ${\gamma }_{I\to I}$, meaning that the intra-layer network effects are stronger for trade shocks than for financial shocks. The network multiplier ${\gamma }_{T\to I}$ is negative, indicating that trade shocks can produce an increase in the incurrence in liabilities, probably to compensate the revenue reduction from exports.

The network multipliers ${\gamma }_{\ell \to \ell ^{\prime} }$ may be used to infer the systemic impact of a country hurt by a combined or single-layer shock, given its relative magnitude in each layer. If we assume that the systemic impact generated by an initial shock in both financial and trade layers, characterized by (*α*, *β*), is comparable to the sum of the systemic impacts of a trade shock with *α*, and a financial shock with *β*, then one can estimate the expected impact as

7 $$(\begin{array}{c}{{\mathcal{S}}}_{i}^{T}(\alpha ,\beta )\\ {{\mathcal{S}}}_{i}^{I}(\alpha ,\beta )\end{array})\simeq (\begin{array}{cc}{\gamma }_{T\to T} & {\gamma }_{I\to T}\\ {\gamma }_{T\to I} & {\gamma }_{I\to I}\end{array})(\begin{array}{c}{\mathcal{I}}_{i}^{T}(\alpha )\\ {\mathcal{I}}_{i}^{I}(\beta )\end{array})$$

**Figure 5:**
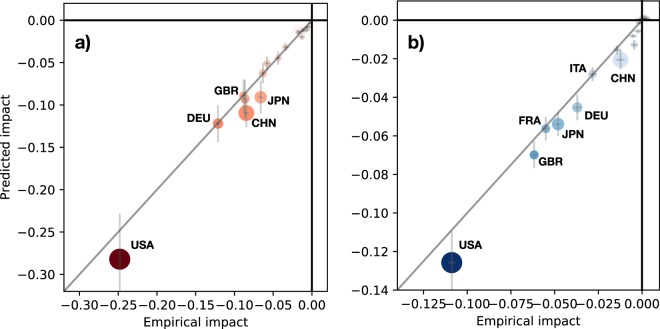
Expected systemic impact versus systemic impact obtained by numerical simulations on trade (**a**) and investment (**b**) of each country *i* belonging to the *G*_20_ group, originated by an initial shock with $\alpha =-\,0.3$, $\beta =-\,0.5$. The size of dots is proportional to their GDP, color proportional to ${{\mathcal{S}}}_{i}^{\ell }$ (red for $\ell =T$, blue for $\ell =I$). Uncertainties are represented by grey crosses.

Figure 5 shows a comparison between the expected impact on trade (Fig. 5a) and investment (Fig. 5b), as derived from Eq. (7), and the systemic impact obtained by numerical simulations, originated by an initial shock with $\alpha =-\,0.3$, $\beta =-\,0.5$. One can see that, by taking into accounts the statistical error on the systemic impact and the uncertainty on the network multipliers ${\gamma }_{\ell \to \ell ^{\prime} }$, expected and numerical impacts are actually very close. Thus, Eq. (7) allows us to infer the systemic impact of a country, given the initial shock (see SM for different values of *α*, *β*), at least for large economies.

Finally, Figs 5 and S11 show clearly that the systemic impact of a country does not only depend on its GDP, and may be significantly different for trade or investment. The country with the largest systemic impact on the rest of the world is by far the United States, with respect to both trade and investment. However, the next countries with the largest impact on investment are the United Kingdom and Japan, while Germany and China have the next largest impact on trade. Note that China, with the second largest GPD, has an expected impact on global investment ten times smaller than the United States.

## Discussion

Estimating the global effects of economic crises remains a major challenge that we have to solve to advance in their prevention and control. We have proved here that a modeling strategy combining a multilayer network approach with inter-country and intra-country contagion dynamics is useful to stress-test the systemic vulnerability of individual countries, that can act as absorbers or amplifiers and of the world economy to propagating shocks. At the large scale, the simple linear relation between the relative magnitude of a shock in a country and its impact on the global system is surprising, since the strength of internal contagion is country-dependent and the inter-country propagation dynamics is non-linear. Interestingly, this systemic impact is associated to intra-layer and inter-layer network multipliers, that are independent of the magnitude of the initial shock.

It is important to remark that our modeling framework has several well-known limitations. Financial data are still scarce for specific economies and often show inconsistency, caused for instance by tax heavens^42^. The missing information should then be estimated at the risk of adding noise coming from the estimation methodologies. On the other hand, our stress-test model represents a solid but first step towards a more sophisticated quantitative framework. For instance, we did not take into account the possible variation of optimal decision rules of economic agents as a response to policy change^45^ and, in order to minimize the number of assumptions, the complexity of the economic structure of a country is neglected. Furthermore, there might be several sources of endogeneity in determining internal pass-through coefficients through Eq. (2), such as omitted variables (e.g., GPD variation), which may lead to biased estimations of the parameters^46^.

Nevertheless, our approach aims at overcoming more serious limitations in the current modeling approach of global shock propagation, mostly based on threshold models, in which a node’s failure triggers a cascade dynamics. Even if the complete collapse of a financial institution has been empirically observed several times, the default of one or more countries, implying the complete stop of trade and financial flows, seems a very unrealistic assumption. Finally, the linearity assumed in the intra-country phase of the shock propagation (another limitation, yet common in standard econometrics) is at least partially compensated by the non-linearity of the inter-country phase, originated from the repeated spreading dynamics.

One natural spinoff of our work would be to analyze how the GTI multiplex network topology, intra-layer pass-through coefficients, and network multipliers evolve in time to compare pre- and post-crisis scenarios. Future work should also be devoted to consider the effects of supply-side shocks, in which the epicenter country does not reduce its imports from other countries, but rather it reduces exports to its trading partners, comparing this scenario with demand-side shocks addressed in this work. In the long run, we hope that our network-based macroeconomic approach to the propagation of shocks could be enriched to contribute to the detection of early warning signals, as well as suggesting regulatory strategies to prevent the social, economic, and ecological cost of crises.

## Methods

Here we describe the empirical data used in the paper, available through motivated request to the authors, and the estimation of the internal pass-through coefficients of the shock propagation model.

### Empirical data

Our work relies on different data sources, described in details in the SM, and summarized here. The investment layer of the GTI network is reconstructed by using the bilateral matrix of cross-border financial positions between countries, as reported in ref. ^42^. Bilateral data disclosing financial exposures are scarce. However, the Coordinated Portfolio Investment Survey (CPIS) annually conducted by the IMF reports data of cross-border positions of portfolio securities between countries. Portfolio securities represent the largest fraction of cross-border investment positions, that include also direct investments and banking sector positions^37^. We consider cross-border portfolio investment positions between two countries as a proxy of the strength of their financial interactions. While portfolio investments account only for a part of a country’s financial exposures, we choose to focus on them for two reasons: i) data regarding portfolio investments are the more complete and reliable available, ii) portfolio investments can be considered short term as compared with other financial exposures, e.g. foreign direct investments, and thus are more appropriate to describe shock propagation. Note that, since CPIS data are biased because of offshore tax heavens, here we considered the data sets compiled in ref. ^42^, which completed CPIS data, as detailed in the SM. The trade layer of the GTI multiplex network is reconstructed by using the United Nations Commodity Trade Statistics Database^41^, also used and described in ref. ^18^. The multivariate regression model, described by Eq. (2), is informed by the time series of exports, imports, incurrence of liabilities, and acquisition of assets, as recorded by the IMF. We considered yearly data, from 1980 to 2015. We exclude global recession periods from the time series, i.e. years 1982, 1991, and 2009^47^.

### Estimation of internal pass-through coefficients

We estimate trend terms, internal pass-through coefficients, and noise terms in Eq. (2) for each country by calculating variances and co-variances of the four time series {*dX*, *dM*, *dA*, *dL*}, extracted from annual data recorded by the IMF, as described in SM. Some observations are in order. First, the model assumes that there are no lags between exports/liabilities revenues and imports/assets payments. Second, one can de-trend the relations described by (2) by setting trend terms equal to zero in the shock propagation dynamics, ${c}_{A}={c}_{M}=0$. Finally, note that correlations between terms *dM* and *dA* are directly related to the correlation between noises *ε*_1_ and *ε*_2_, as $\langle dMdA\rangle ={\sigma }_{{\varepsilon }_{1}{\varepsilon }_{2}}^{2}$, see SM.

## Supplementary information

Supplementary Material

## References

[1] Allen, F. & Gale, D. *Understanding Financial Crises*, https://EconPapers.repec.org/RePEc:oxp:obooks:9780199251421 (Oxford University Press, 2009).

[2] Lane, P. & Milesi-Ferretti, G. M. The external wealth of nations: Measures of foreign assets and liabilities for industrial and developing countries. Economic Papers, Trinity College Dublin, Economics Department, https://EconPapers.repec.org/RePEc:tcd:tcduee:20014 (2001).

[3] PAVLOVA ANNA, RIGOBON ROBERTO (2008). The Role of Portfolio Constraints in the International Propagation of Shocks. Review of Economic Studies.

[4] Stiglitz, J. Risk and global economic architecture: Why full financial integration may be undesirable. *American Economic Review* **100**, 388–92, https://EconPapers.repec.org/RePEc:aea:aecrev:v:100:y:2010:i:2:p:388-92 (2010).

[5] Canova Fabio, Marrinan Jane (1998). Sources and propagation of international output cycles: Common shocks or transmission?. Journal of International Economics.

[6] Rigobon, R. On the measurement of the international propagation of shocks. Working Paper 7354, National Bureau of Economic Research, http://www.nber.org/papers/w7354 (1999).

[7] Lumsdaine RL, Prasad ES (2003). Identifying the common component of international economic fluctuations: A new approach*. The Economic Journal.

[8] Newman MEJ (2010). Networks: An introduction.

[9] Bakshy, E., Rosenn, I., Marlow, C. & Adamic, L. The role of social networks in information diffusion. In *Proceedings of the 21st International Conference on World Wide Web*, WWW ’12, 519–528, 10.1145/2187836.2187907 (ACM, New York, NY, USA, 2012).

[10] Pastor-Satorras R, Castellano C, Van Mieghem P, Vespignani A (2015). Epidemic processes in complex networks. Rev. Mod. Phys..

[11] Serrano MA, Boguñá M (2003). Topology of the world trade web. Phys. Rev. E.

[12] Garlaschelli D, Loffredo MI (2004). Fitness-dependent topological properties of the world trade web. Phys. Rev. Lett..

[13] Serrano MÁ, Boguñá M, Vespignani A (2007). Patterns of dominant flows in the world trade web. Journal of Economic Interaction and Coordination.

[14] Hidalgo C. A., Klinger B., Barabasi A.-L., Hausmann R. (2007). The Product Space Conditions the Development of Nations. Science.

[15] Fagiolo G, Reyes J, Schiavo S (2009). World-trade web: Topological properties, dynamics, and evolution. Phys. Rev. E.

[16] Serrano, M. A., Garlaschelli, D., Boguñá, M. & Loffredo, M. The world trade web: Structure, evolution and modeling. In Caldarelli, G. (ed.) *Complex Networks*, Encyclopedia of Life Support Systems (EOLSS) (Eolss Publishers, Oxford, UK, 2010).

[17] De Benedictis L, Tajoli L (2011). The world trade network. The World Economy.

[18] Garca-Pérez G, Boguñá M, Allard A, Serrano MÁ (2016). The hidden hyperbolic geometry of international trade: World trade atlas 1870–2013. Scientific Reports.

[19] Lee Kyu-Min, Yang Jae-Suk, Kim Gunn, Lee Jaesung, Goh Kwang-Il, Kim In-mook (2011). Impact of the Topology of Global Macroeconomic Network on the Spreading of Economic Crises. PLoS ONE.

[20] Kireyev, A. & Leonidov, A. Network effects of international shocks and spillovers. IMF Working Papers 15/149, International Monetary Fund, https://EconPapers.repec.org/RePEc:imf:imfwpa:15/149 (2015).

[21] Lee K-M, Goh KI (2016). Strength of weak layers in cascading failures on multiplex networks: case of the international trade network. Scientific Reports.

[22] Bardoscia M, Battiston S, Caccioli F, Caldarelli G (2017). Pathways towards instability in financial networks. Nature Communications.

[23] Caccioli F, Barucca P, Kobayashi T (2018). Network models of financial systemic risk: a review. Journal of Computational Social Science.

[24] Acemoglu, D., Ozdaglar, A. & Tahbaz-Salehi, A. Systemic risk and stability in financial networks. Working Paper 18727, National Bureau of Economic Research, http://www.nber.org/papers/w18727 (2013).

[25] Battiston Stefano, Caldarelli Guido, May Robert M., Roukny Tarik, Stiglitz Joseph E. (2016). The price of complexity in financial networks. Proceedings of the National Academy of Sciences.

[26] Cont, R., Moussa, A. & Santos, E. B. Network structure and systemic risk in banking systems. Post-Print, HAL, https://EconPapers.repec.org/RePEc:hal:journl:hal-00912018 (2013).

[27] Battiston S, Puliga M, Kaushik R, Tasca P, Caldarelli G (2012). Debtrank: Too central to fail? financial networks, the fed and systemic risk. Scientific Reports.

[28] Chinazzi, M. & Fagiolo, G. Systemic Risk, Contagion, and Financial Networks: A Survey. LEM Papers Series 2013/08, Laboratory of Economics and Management (LEM), Sant’Anna School of Advanced Studies, Pisa, Italy, https://ideas.repec.org/p/ssa/lemwps/2013-08.html (2013).

[29] Espinosa-Vega MA, Solé J (2011). Cross-border financial surveillance: a network perspective. Journal of Financial Economic Policy.

[30] Gai Prasanna, Kapadia Sujit (2010). Contagion in financial networks. Proceedings of the Royal Society A: Mathematical, Physical and Engineering Sciences.

[31] Bargigli L, di Iasio G, Infante L, Lillo F, Pierobon F (2015). The multiplex structure of interbank networks. Quantitative Finance.

[32] Brummitt CD, Kobayashi T (2015). Cascades in multiplex financial networks with debts of different seniority. Phys. Rev. E.

[33] Minoiu Camelia, Reyes Javier A. (2013). A network analysis of global banking: 1978–2010. Journal of Financial Stability.

[34] Joseph AC, Joseph SE, Chen G (2014). Cross-border portfolio investment networks and indicators for financial crises. Scientific Reports.

[35] Castrén, O. & Kavonius, I. K. Balance Sheet Interlinkages and Macro-Financial Risk Analysis in the Euro Area. Working Paper Series 1124, European Central Bank, https://ideas.repec.org/p/ecb/ecbwps/20091124.html (2009).

[36] Hale, G., Kapan, T. & Minoiu, C. Crisis Transmission in the Global Banking Network. IMF Working Papers 16/91, International Monetary Fund, https://ideas.repec.org/p/imf/imfwpa/16-91.html (2016).

[37] Korniyenko, Y., Patnam, M., del Rio-Chanon, R. M. & Porter, M. A. Evolution of the Global Financial Network and Contagion: A New Approach. IMF Working Papers 18/113, International Monetary Fund, https://ideas.repec.org/p/imf/imfwpa/18-113.html (2018).

[38] Boccaletti S (2014). The structure and dynamics of multilayer networks. Physics Reports.

[39] Poledna Sebastian, Molina-Borboa José Luis, Martínez-Jaramillo Serafín, van der Leij Marco, Thurner Stefan (2015). The multi-layer network nature of systemic risk and its implications for the costs of financial crises. Journal of Financial Stability.

[40] De Domenico M, Granell C, Porter MA, Arenas A (2016). The physics of spreading processes in multilayer networks. Nat Phys.

[41] United nations commodity trade statistics database (un comtrade), https://comtrade.un.org/db/ (2018).

[42] Zucman G (2013). The missing wealth of nations: Are europe and the u.s. net debtors or net creditors?*. The Quarterly Journal of Economics.

[43] Benguria, F. & Taylor, A. M. After the panic: Are financial crises demand or supply shocks? Evidence from international trade. Working Paper 25790, National Bureau of Economic Research, http://www.nber.org/papers/w25790 (2019).

[44] Gourinchas, P.-O. & Rey, H. International financial adjustment. Working Paper 11155, National Bureau of Economic Research, http://www.nber.org/papers/w11155 (2005).

[45] Lucas, R. Econometric policy evaluation: A critique. *Carnegie-Rochester Conference Series on Public Policy* **1**, 19–46, https://EconPapers.repec.org/RePEc:eee:crcspp:v:1:y:1976:i::p:19-46 (1976).

[46] Wooldridge, J. *Introductory Econometrics: A Modern Approach (with Economic Applications*, *Data Sets*, *Student Solutions Manual Printed Access Card)*, 4 edn. (South-Western College Pub, 2008).

[47] *World Economic Outlook: Crisis and Recovery* (International Monetary Fund, April 2009).

