# Supplementary material for: The interconnected wealth of nations: Shock propagation on global trade-investment multiplex networks
Source: Sci Rep. 2019 Sep 11;9:13079. doi: 10.1038/s41598-019-49173-2 (PMC6739386; doi:10.1038/s41598-019-49173-2)
Supplement: Supplementary file 1 — Supplementary Material [file 41598_2019_49173_MOESM1_ESM.pdf]

Supplementary Material for the paper:
"The interconnected wealth of nations:
Shock propagation on global trade-investment multiplex networks"

Michele Starnini,[1,*] Marián Boguñá,[2,3] and M. Ángeles Serrano[3,3,4,†]

[1]*Data Science Laboratory, ISI Foundation, Torino, Italy*
[2]*Departament de Física de la Matèria Condensada, Universitat de Barcelona, Martí i Franquès 1, 08028 Barcelona, Spain*
[3]*Universitat de Barcelona Institute of Complex Systems (UBICS), Universitat de Barcelona, Barcelona*
[4]*ICREA, Pg. Lluís Companys 23, E-08010 Barcelona, Spain*

[*] Corresponding author: michele.starnini@gmail.com
[†] Corresponding author: marian.serrano@ub.edu

I. EMPIRICAL DATA SETS DESCRIPTION

In this section, we describe the empirical data sets used in the paper. Our work relies on the following data sources.

- The **Coordinated Portfolio Investment Survey (CPIS)** reports bilateral data on cross border portfolio investments between pairs of countries. The survey is conducted annually by the International Monetary Fund (IMF), started in 2001, and it distinguishes between equity and debt securities. Participation in the survey by countries is voluntary. The survey is conducted by asking a creditor country i for its cross-border assets a_{ij}, issued by a debtor country j. The asset a_{ij} is equivalent to a liability l_{ji}, issued by country j, owned by country i. The sum of assets owned by i is $A_i = \sum_j a_{ij}$, and the sum of its liabilities reads $L_i = \sum_j l_{ij}$. Therefore, in the CPIS liabilities are derived for both reporting and non-reporting countries. If all countries reported in the CPIS, the resulting cross-border (portfolio) investment network would be a fully connected graph. For the year 2008, a total of 73 creditors (excluding important economies, such as China and oil exporters) reported on more than 200 debtors. Note that CPIS reports financial data only regarding portfolio investment, not including other components of the financial account (FA), such as directed investment (DI), financial derivatives (FD) or other investment (OI).

  The Cross-Border Investment Network (CBIN) is reconstructed from the bilateral matrix of cross-border financial position between countries, with data provided by the CPIS. Since the CPIS data are incomplete and present problem of internal incoherence, underestimating the net foreign asset positions of rich countries because of offshore tax havens, here we considered the data sets compiled in Ref. [2], that completed CPIS data in order to recover internal coherence, for years from 2001 till 2008. Note that even in this case, for some countries, mainly Luxemburg, Cayman Islands and Ireland, the liabilities-side reported are largely incorrect, due to massive unreported investment in the financial industry of these countries, that lately re-direct such investment toward other countries. Reserves held by central banks and International organization are aggregated together, represented under the label SEFER+SSIO. We exclude this node from the network.

- The World Trade Web (WTW) is reconstructed from the data set compiled in Ref. [1]. In the WTW, each node represent a country, and a the weight w_{ij} of the direct link from i to j represents the amount of exports (in 2006 dollars) from country i to country j. Since we are interest in a multiplex representation combining the WTW with the CBIN, also in this case we consider years from 2001 to 2008.

- The shock propagation model, described in the main text and in more details in Section III, is informed by the time series of exports, imports, incurrence of liabilities, and acquisition of assets. Such time series are reported by the IMF as aggregated data (i.e. a single country vs rest of the world) of the balance of payments (BOP) for most countries. We informed the shock propagation model by yearly data from 1980 to 2015, excluding global recession periods from the time series, i.e. years 1982, 1991, and 2009. Depending on the country and time period, different level of detail is available (e.g. for South Africa, 1992, it is available the FA, but not its single components, such as DI or PI). Unfortunately, data of the BOP recorded by the IMF are generally not broken down to single counterparts, that is, bilateral data (i.e. a country i vs another country j) are not reported by the IMF. The only data source for bilateral financial data is the CPIS. On the contrary, bilateral data regarding trade in goods have been collected by different sources, e.g. COMtrade.

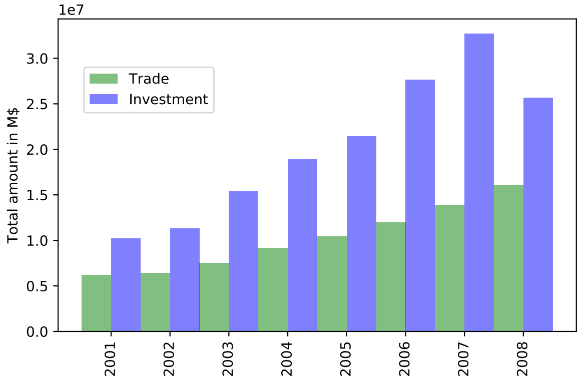

Figure 1: Global value of trade and investment from 2001 to 2008

II. TOPOLOGY OF GTI MULTIPLEX NETWORKS

In this section, we describe how we reconstruct global trade-investment (GTI) networks, and discuss their topological properties. International economic transactions, summarized by the balance of payments (BOP), can be represented as a multiplex network: each node represents a country, and different accounts (such as current and financial account) are described by different layers. Since we are interested in shock propagation, we reconstruct a network of "vulnerabilities" between countries, in which a shock can be transmitted by two main channels, trade and investment, represented by different layers of a multiplex network. From a practical point of view, a multiplex representation of global macroeconomic networks is obtained by coupling the WTW, forming the T layer, with the CBIN, representing the I layer.

By considering the data sets available in Ref. [2], GTI networks can be reconstructed for each year between 2001 and 2008. Figure 1 shows the time evolution of the global value of traded goods W_T and investment positions W_I, as defined by Eq. (1) of the main text. One can see that both quantities increase in time, but at different paces: while before the year 2000 total investment and trade were comparable, between 2001 and 2007 the total investment increased by more than three times, before decreasing in 2008 due to the financial crisis. The slower growth of global trade may be rooted in real-economy constraints, such as production capacity or shipping. The volume and exponential growth of global investment, on the contrary, demonstrates the need of including the financial layer in the study of international shock propagation.

GTI networks are directed, weighted, multiplex networks. Nodes represent countries, links in the T layer represent exports/imports, links in the I layer represent portfolio investments (that can be equity and/or debt) between countries, committed by both public and private actors. The weight w_{ij}^{ℓ} thus represents the weight from node i to node j in layer ℓ. In Table I, we report some properties of the network obtained by data corresponding to the year 2005, which we use in the main paper. Both layers are weakly but not strongly connected, with less than half of links being bidirectional and a relatively small reciprocity value ρ. Both layers are quite dense, with a large average degree $\langle k \rangle$, and rather homogeneous degree distribution $P(k)$.

Despite the fact that degrees are quite homogeneously distributed, weights in both layers are very heterogeneous, as revealed by the broad tailed form of the weight distribution, shown in Fig. 2. The weight distributions of both layers, $P_T(w)$ and $P_I(w)$, are compatible with power-law forms, $P_\ell(w) \sim w^{-\gamma_\ell}$, with slightly different exponents $\gamma_I \simeq 1.36$ and $\gamma_T \simeq 1.5$. The out- and in-strength of node i, defined as $s_{i,\ell}^{out} = \sum_j w_{ij}^{\ell}$ and $s_{i,\ell}^{in} = \sum_j w_{ji}^{\ell}$, represent the total assets held and liabilities issued by a country i for the investment layer, $\ell = I$, while they represent the total of exports and imports of the same country i for $\ell = T$. Figure 2 shows that the distributions $P_\ell(s)$ are also heavy tailed, although noisy, due to the small size of the network. All distributions are compatible with power-law forms, $P_\ell(s) \sim s^{-\gamma_\ell}$, with exponents $\gamma_\ell \in [1.1, 1.3]$.

Table I: Some topological properties of GTI multiplex: number of nodes N, number of directed E_ℓ and overlapped E_O edges, total weight W_ℓ (expressed in 10^{12} dollars), in trade $\ell = T$ and investment $\ell = I$ layers.

N	E_T	E_I	E_O	W_T	W_I
186	12540	4499	3617	10.4	21.4

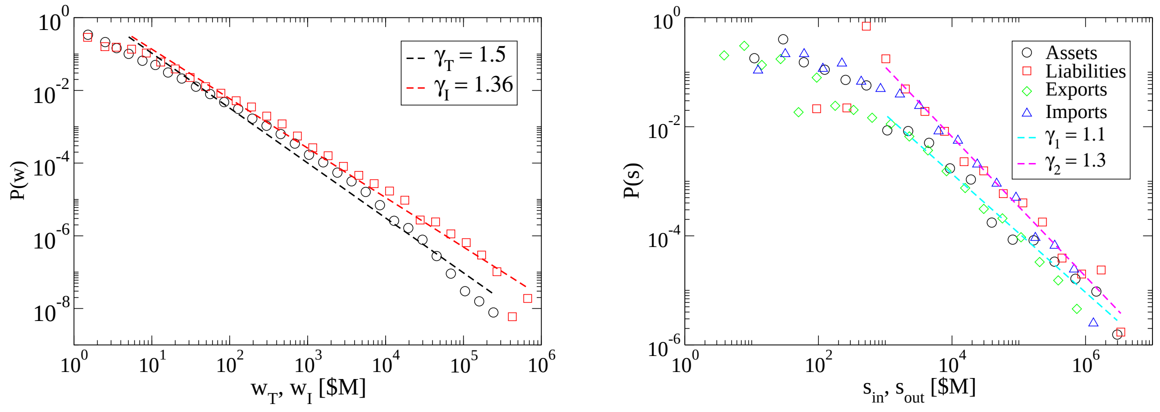

Figure 2: Probability distribution of weights w_{ij}^{ℓ} (left), and in- and out-strength s_j^{ℓ} for layers $\ell = T$ and $\ell = I$ of the multiplex macroeconomic networks. Power law functional forms $P(w) \sim w^{-\gamma}$ and $P(s) \sim s^{-\gamma}$ are drawn as a reference.

III. SIR DYNAMICS OF THE SHOCK PROPAGATION FROM DISTRESSED TO NEIGHBORING COUNTRIES

In this section, we describe the details of the contagion dynamics of the shock propagation model, giving a concrete example to show how the economic distress spreads over the network. First of all, let us fix the notation. Each link between two nodes i and j of the multiplex network is characterized by four macroeconomic quantities: goods exported from i to j, x_{ij}, and goods imported from i to j, $m_{ij} \equiv x_{ji}$, in the T layer, assets held by i and issued by j, a_{ij}, and liabilities issued by i and held by j, $l_{ij} \equiv a_{ji}$, in the I layer. In the same way, each node i is characterized by four aggregated quantities: its total exports X_i, total imports M_i, total assets held A_i, total liabilities issued L_i. The dynamics of the network is represented by allowing these quantities to vary in time, adding explicitly the time-dependency: $x_{ij}(t)$, $a_{ij}(t)$, $M_i(t)$, $L_i(t)$, etc. The notation $\delta Y_i(t) \equiv \frac{Y_i(t)-Y_i(t-1)}{Y_i(t-1)}$ stands for the relative variation of $Y_i(t)$ in time, $Y_i = \{X_i, M_i, A_i, L_i\}$, where time t is accounted by discrete time steps in the shock propagation process, $t = 0, 1, \ldots, t_{end}$. Equivalently, $\delta y_{ij}(t)$ represents the relative variation of quantity $y_{ij} = \{x_{ij}, m_{ij}, a_{ij}, l_{ij}\}$ in time.

The contagion dynamics of the shock propagation model is simulated by means of a a Susceptible-Infected-Recovered (SIR) model, that allows to properly address reverberation and second order effects. It is important, indeed, to consider back and forth effects, since the epicenter country i, as well as any other node, can be hit back by the distress propagation. A node i, indeed, propagates the distress to a neighbor j through links $x_{ji}(t)$ and $l_{ji}(t)$, and subsequently, node j propagates its distress to all his neighbors, including node i itself, though links $x_{ij}(t)$ and $l_{ij}(t)$. Moreover, each node that already propagated the distress will be further hit back by other neighboring nodes, as soon as the distress reaches them.

In the SIR model, at each time t, each node i can be in one of three states:

- **Susceptible,** $s_i(t) = S$**:** the country can receive distress from its neighbors, $\delta X_i(t) \neq 0$, $\delta L_i(t) \neq 0$, but it has not propagated it yet, $\delta M_i(t) = \delta A_i(t) = 0$;
- **Infected,** $s_i(t) = I$**:** the country propagates the distress accumulated to his neighbor by applying Eq. (2) of the main text, and it is characterized by $\delta M_i(t) \neq 0$ and $\delta A_i(t) \neq 0$;
- **Recovered or inactive,** $s_i(t) = R$**:** the country can receive distress from its neighbors, $\delta X_i(t) \neq 0$, $\delta L_i(t) \neq 0$, but it does not propagate it anymore.

The SIR dynamics can be summarized as follows. The following two steps are repeated in loop until no more infected node are present:

- **Step 1**, each node i in an infected state at time t, $s_i(t) = I$ (with $\delta M_i(t) \neq 0$ or $\delta A_i(t) \neq 0$) propagates the distress to all its neighbors (regardless of their status) and it becomes inactive immediately after, $s_i(t+1) = R$.

- **Step 2**, each node i in a susceptible state at time t, $s_i(t) = S$, with $\delta X_i(t) \neq 0$ or $\delta L_i(t) \neq 0$ (thus each node that received distress from any neighbor), applies Eq. (2) and becomes infected at time $t+1$, $s_i(t+1) = I$. After applying Eq. (2), he sets $\delta X_i(t+1) = \delta L_i(t+1) = 0$.

Step one and step two are repeated in loop until at some time t^* each node i will be in a susceptible (with $\delta X_i(t^*) = \delta L_i(t^*) = 0$) or inactive state (with $\delta X_i(t^*) \neq 0$ or $\delta L_i(t^*) \neq 0$).

It is worth to describe the distress propagation dynamics over the networks by means of a concrete example. Let us assume that at $t = 0$, a shock originates in an epicenter country i. which, at time $t = 1$, reduces its imports by a factor α_1 and its investment in foreign assets by a factor β_1. For the sake of clarity in what follows, here we consider $|\alpha_1| > 0$ and $|\beta_1| > 0$ as the absolute value of the initial negative variation, while in the main text the initial variation are indicated by α and β and can have any sign.

- At $t = 1$, node i is in a infected state with $M_i(t = 1) = (1 - \alpha_1)M_i(t = 0)$ and $A_i(t = 1) = (1 - \beta_1)A_i(t = 0)$, while all other nodes j are susceptible, $s_j(t = 1) = S \quad \forall j$. Since reductions in imports and assets are distributed proportionally among the neighboring nodes, the weight of each link from j to i is reduced by the same factor α_1 and β_1, that is, $m_{ij}(t = 1) = (1 - \alpha_1)m_{ij}(t = 0)$ and $a_{ij}(t = 1) = (1 - \beta_1)a_{ij}(t = 0)$. Since imports from country i to country j are equal to exports from country j to country i, each neighbor j reduces its export to country i as $x_{ji}(t = 1) = (1 - \alpha_1)x_{ji}(t = 0)$, and the same applies for liabilities, $l_{ji}(t = 1) = (1 - \beta_1)l_{ji}(t = 0)$. This implies that each neighbor j is forced to reduce its total exports by a different factor α_1^j, $X_j(t = 1) = (1 - \alpha_1^j)X_j(t = 0)$, and its total liabilities by a different factor β_1^j, $L_j(t = 1) = (1 - \beta_1^j)L_j(t = 0)$, or equivalently $\delta X_1^j = -\beta_1^j$ and $\delta L_1^j = -\alpha_1^j$, in the notation of the main text. Factors $\alpha_1^j \leq \alpha_1$ and $\beta_1^j \leq \beta_1$ depend on how important is country i as economic partner for country j. In the case limit of node i being the only neighbor of node j in both layers, it holds $\alpha_1^j = \alpha_1$ and $\beta_1^j = \beta_1$. At this point, all infected nodes (in this case, only the epicenter country) have propagated the economic distress, thus step one of the loop is concluded.

- In the next step, $t = 2$, the epicenter country i is set to a inactive state, $s_i(t = 2) = R$, and all neighboring nodes j in a susceptible state that received the distress, with $\delta X_j(t = 1) \neq 0$ or $\delta L_j(t = 1) \neq 0$, become infected, $s_j(t = 2) = I$. The variations of imports and asset investment of each country j, $\delta M_j(t = 2)$ and $\delta A_j(t = 2)$ are obtained through Eq. (2) of the main text, as a function of the variation of export and liabilities incurrence in the previous time step, $\delta M_j(t = 2) = f(\delta X_j(t = 1), \delta L_j(t = 1))$ and $\delta A_j(t = 2) = f(\delta X_j(t = 1), \delta L_j(t = 1))$. These variations depend on the set of propagation coefficients c_{MX}, c_{ML}, c_{AX}, and c_{AL}, and the noise terms. Immediately after applying Eq. (2) of the main text, each country j sets $\delta X_j(t = 2) = \delta L_j(t = 2) = 0$, to receive the next round of economic distress from his neighbors. At this point, all susceptible nodes have applied Eq. (2), thus step two of the loop is concluded.

- We now repeat step one: each country j in a infected state, $s_j(t = 2) = I$ propagates the variations $\delta M_j(t = 2)$ and $\delta A_j(t = 2)$ proportionally to each neighbor k through both trade and investment links, $\delta m_{jk}(t = 2) = \delta M_j(t = 2)$ and $\delta a_{jk}(t = 2) = \delta A_j(t = 2)$. Note that the distress is propagated to all neighbors k, including the infected nodes (all the neighbors j of the epicenter country) and the inactive nodes (only the epicenter country i). The total exports and liabilities of node k are thus reduced by a different factors α_2^k and β_2^k, $\delta X_k(t = 2) = -\alpha_2^k$ and $\delta L_k(t = 2) = -\beta_2^k$. After all infected nodes (all neighbors j of the epicenter country) have propagated the economic distress, step one of the loop is concluded.

- Step two is repeated: At time $t = 3$, all nodes j previously infected become inactive, $s_j(t = 3) = R$, and nodes k that received the distress, $\delta X_k(t = 2) \neq 0$ or $\delta L_k(t = 2) \neq 0$ that were susceptible, $s_k(t = 2) = S$, become infected. That is, $s_k(t = 3) = I$ only if $s_k(t = 2) = S$ and $(\delta X_k(t = 2) \neq 0 \lor \delta L_k(t = 2) \neq 0)$. All nodes that become infected apply Eq. (2), obtaining variation of imports and asset investment $\delta M_k(t = 3)$ and $\delta A_k(t = 3)$ from variations $\delta X_k(t = 2)$ and $\delta L_k(t = 2)$ of the previous time step. Afterwords, each infected node k sets $\delta X_k(t = 3) = \delta L_k(t = 3) = 0$, to receive the next round of economic distress from its neighbors, and step two is concluded again.

Step one and step two are repeated in loop until at some point $t = t^*$, each node i will be in a susceptible (with $\delta X_i(t^*) = \delta L_i(t^*) = 0$) or inactive state (with $\delta X_i(t^*) \neq 0$ or $\delta L_i(t^*) \neq 0$). At this point, the SIR dynamics is concluded, and the economic distress has propagated through all the network. Note that each node i propagates the distress accumulated by applying Eq. (2) of the main text at most one time.

It is important to note that at $t = t^*$, each inactive node i is characterized by $\delta X_i(t^*) \neq 0$ or $\delta L_i(t^*) \neq 0$, meaning that it is affected by second order effects of the contagion, that should be taken into account. For this reason, we repeat the whole SIR dynamics, by using the distress accumulated by inactive nodes as initial conditions for the new dynamics. Each inactive node i at time t^*, $s_i(t^*) = R$, becomes infected at time $t^* + 1$, $s_i(t^* + 1) = I$, thus it applies Eq. (2), obtaining new variations in imports and asset investment, $\delta M_i(t^* + 1)$ and $\delta A_i(t^* + 1)$. By repeating the SIR dynamics, a second contagion wave spreads over the network, until again at some time $t = t^{**}$ each node will be in a susceptible or inactive state. We repeat the SIR dynamics a number n of times, that ensures that the system has reached a steady state. The fact that at each reverberation the distress propagated is smaller ensures that the dynamics converges quickly.

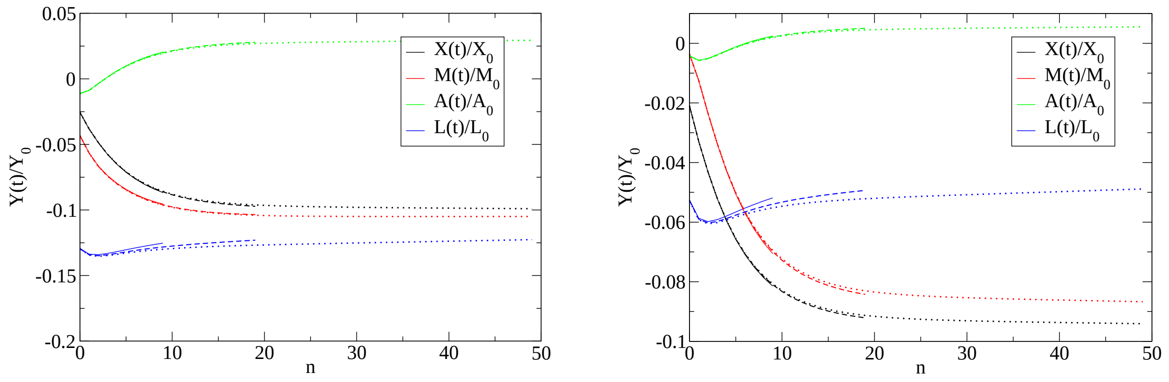

Figure 3: Evolution of exports, imports, assets and liabilities of the UK (left) and Germany (rigth) by using a different number of repetions n of the SIR contagion dynamics. Results averaged over 100 runs.

It is important to note that the duration of the whole contagion dynamics, given by the n repetitions of the SIR dynamics, is a free parameter of the model. However, after a certain number of reverberations the system is stable and does not evolve in time anymore. Figure 3 shows the evolution in time of exports, imports, assets and liabilities, $X_i(t)$, $M_i(t)$, $A_i(t)$, and $L_i(t)$ for two countries, $i=$ UK and $i=$ Germany, with respect to the same quantities at $t=0$, the time at which the shock takes place in a single epicenter country, namely the United States, with initial conditions $\alpha=-0.1$, $\beta=-0.3$. One can see that after $n\simeq 20$ repetition steps the system does not evolve in time anymore. Note also that there are slight differences in the evolution by choosing a different number of repetitions n (represented by continuous and dashed lines in Fig. 3). In the paper we set $n=50$ that ensures that the system has reached a steady state at time T after the shock.

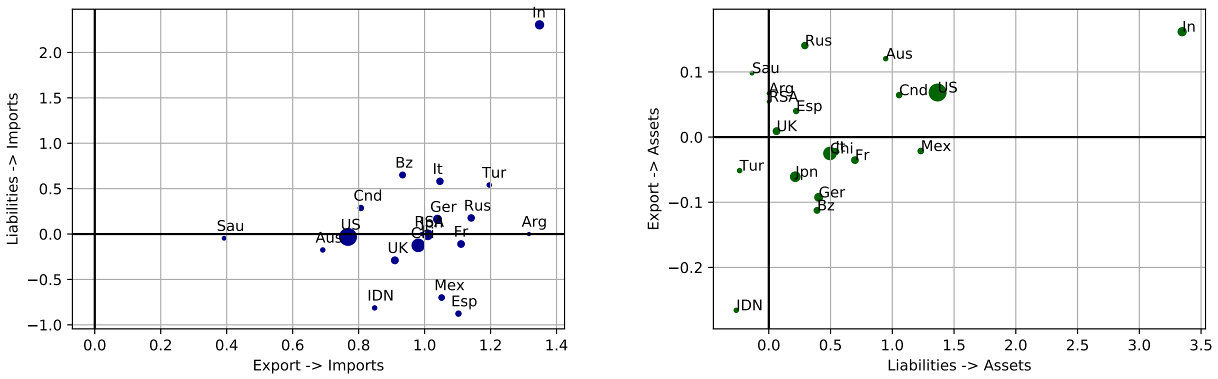

Figure 4: Scatter plots of intra-layer (x-axis) versus inter-layer (y-axis) coefficients. Pass-through coefficients to trade (c_{MX} on the x-axis and c_{ML} on the y-axis), and to investment (c_{AL} on the x-axis and c_{AX} on the y-axis), for the G_{20} group. The size of the countries is proportional to their GDP.

IV. ESTIMATION OF THE PASS-THROUGH COEFFICIENTS OF SHOCK PROPAGATION MODEL

In this section, we describe how we estimate pass-through coefficients of the shock propagation model, and we briefly discuss them. For each country, the trend terms, pass- through coefficients, and noise terms in Eq. (2) of the main text are estimated by calculating variances and co-variances of the four time series $\{dX_t, dM_t, dA_t, dL_t\}$. We consider yearly data from 1980 to 2015, by excluding recession periods (i.e. years 1982, 1991, 2008), as reported by the IMF, see Section I. The variance of the noise terms, $\sigma^2_{\epsilon_1}$ and $\sigma^2_{\epsilon_2}$, incorporates the reliability of the propagation coefficients and trend terms. If data for a country are scarce, with respect to one or several time series, and no clear relation emerges from two macroeconomic variables, then the variance of the corresponding noise term will be large if compared to the coefficient multiplied by the variance of the corresponding variable, e.g. $\sigma^2_{\epsilon_1} \gg c_1 \langle dL^2_t \rangle$. In this case, such country would propagate only noise to the system. For this reason, if for any country, at time t, it holds e.g. $\sigma^2_{\epsilon_1} \geq b_1 \langle dX^2_t \rangle$ or $\sigma^2_{\epsilon_1} \geq c_1 \langle dL^2_t \rangle$, then we set $dM_t = 0$. The same conditions apply for the term dA_t. This might underestimate the spillover effect, but it ensures the system to be stable. We check that the countries that do not fulfill conditions for stability are few.

Figure 4 shows pass-through coefficients for countries belonging to the G_{20} group. The plot on the left shows propagation coefficients c_{MX} (export to import) and c_{ML} (liabilities to import), while the plot on the right shows propagation coefficients c_{AL} (liabilities to assets) and c_{AX} (exports to assets). The major contribution for the propagation of a shock on the GTI multiplex comes from *intra-layer* coefficients acting within the same layer. For many countries, the inter-layer propagation $I \to T$, represented by coefficient c_{ML}, is larger than the propagation $T \to I$, represented by coefficient c_{AX}. For trade, Fig.4 (left), the intra-layer term c_{MX}, representing the dependence between imports and exports, is dominant with respect to the inter-layer term c_{ML}. If we exclude the case of India, it holds $|c_{MX}| > |c_{ML}|$ for all large economies, as expected. The coefficient c_{MX} is bounded between $c_{MX} \in [0, 1.5]$, with most countries having $c_{MX} \simeq 1$, indicating a positive, strong correlation between imports and exports, as previous empirical findings showed. The most notable exception is Saudi Arabia with $c_{MX} \simeq 0.4$, a rich Middle East oil producer, whose imports are known to depend little on revenues from exports and financial assets. The inter-layer term c_{ML} is generally bounded between $c_{ML} \in [-0.75, 0.75]$, indicating that the dependence between a variation in exports and the incurrence in liabilities can be positive or negative, depending on the country. For investment, Fig.4 (right), the intra-layer term c_{AL} is also dominant with respect to the inter-layer term c_{AX}. The coefficient between variation of assets and liabilities is generally positive, also bounded between $c_{AL} \in [0, 1.5]$ (excluding India). The inter-layer coefficient c_{AX} is much smaller, bounded between $c_{ML} \in [-0.3, 0.2]$, indicating that the correlation between a variation in exports and assets acquisition is very weak. The dominance of intra-layer propagation terms, generally positive, is confirmed by simple considerations of balance of payments flows: a negative (positive) variation in export (liabilities) revenues is expected to generate a negative (positive) variation in imports (asset acquisition).

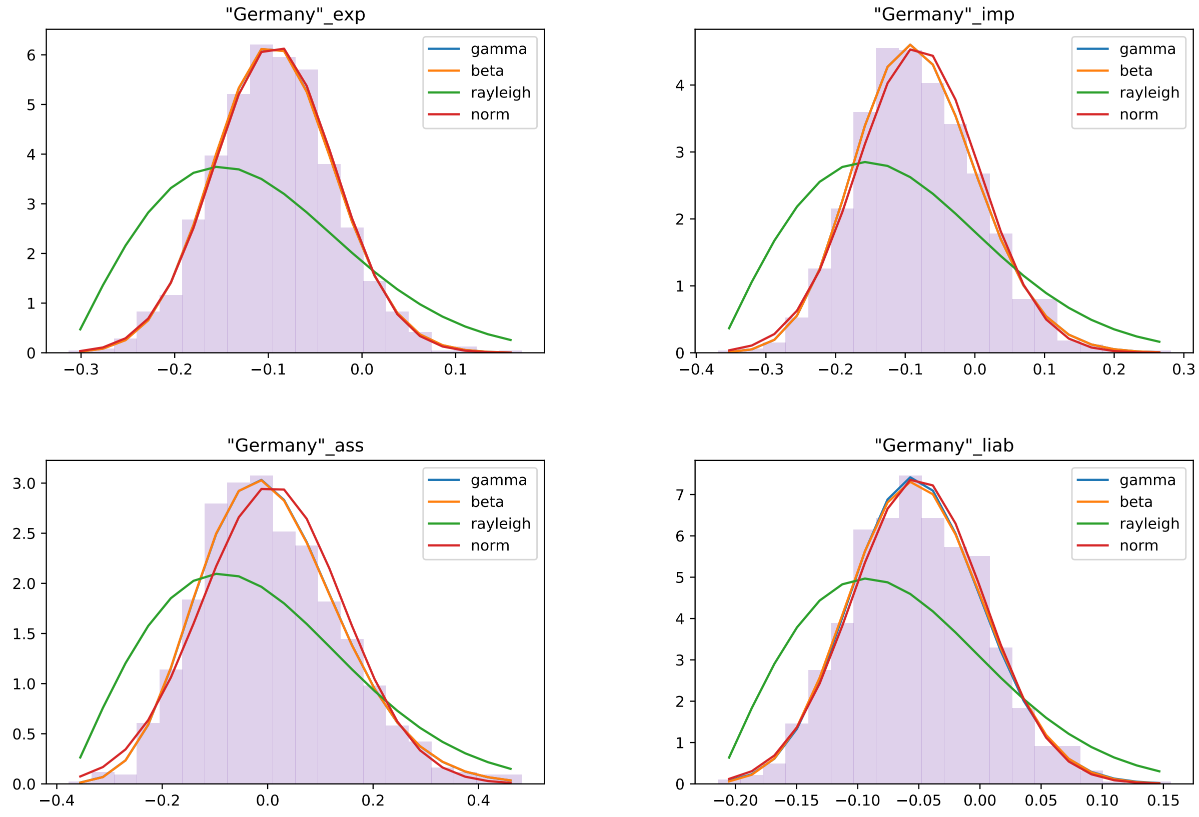

Figure 5: Distributions of the relative variations $\Delta X_i(\alpha,\beta)$ (top, right), $\Delta M_i(\alpha,\beta)$ (top, left), $\Delta A_i(\alpha,\beta)$ (bottom, left), and $\Delta L_i(\alpha,\beta)$ (bottom, right), for parameters $\alpha = -0.1$ and $\beta = -0.3$. Fitting functions gamma, beta, Rayleigh and normal functions are plotted as continuous lines with different colors.

V. VULNERABILITY OF COUNTRIES TO PROPAGATING SHOCKS

The impact of an initial shock characterized by parameters (α,β), in an epicenter country E, to another country i, can be quantified by considering the relative variations $\Delta Y_i(\alpha,\beta,E)$ of each macroeconomic quantity $Y_i = \{X_i, M_i, A_i, L_i\}$ (exports, imports, assets acquisition and liabilities incurrence) of country i, as described by Eq. (3) of the main text. Figure 5 shows the distribution of relative variations $\Delta Y_i(\alpha,\beta,E)$ for $i =$ Germany, obtained by a shock with parameters $\alpha = -0.1$ and $\beta = -0.3$ and epicenter in the United States. We find the best fit of these distribution with some standard fitting function, i.e. gamma, beta, Rayleigh, and normal functions, and extract the average, $\langle \Delta Y_i(\alpha,\beta,E)\rangle$, and the Value-at-Risk, $VaR[\Delta Y_i(\alpha,\beta,E)]$, for $p = 0.05$ of each quantity, defined such that the probability of a negative variation greater than VaR is less than or equal to p while the probability of a loss less than VaR is less than or equal to $1-p$.

Figure 6 shows the average vulnerability of exports, $\langle V_i(X_i)\rangle$ (left plots), and incurrence in liabilities, $\langle V_i(L_i)\rangle$, of each country i with respect to a shock originated in the United States (first row), or in China (second row), or in countries belonging to the EZ (third row), characterized by $\alpha = -0.4$ and $\beta = -0.1$. There maps correspond to Fig. 1 of the main text, which shows the VaR of the same quantities, for the same initial shock. One can see that the average vulnerability shows the same qualitative behavior across different countries of the corresponding VaR.

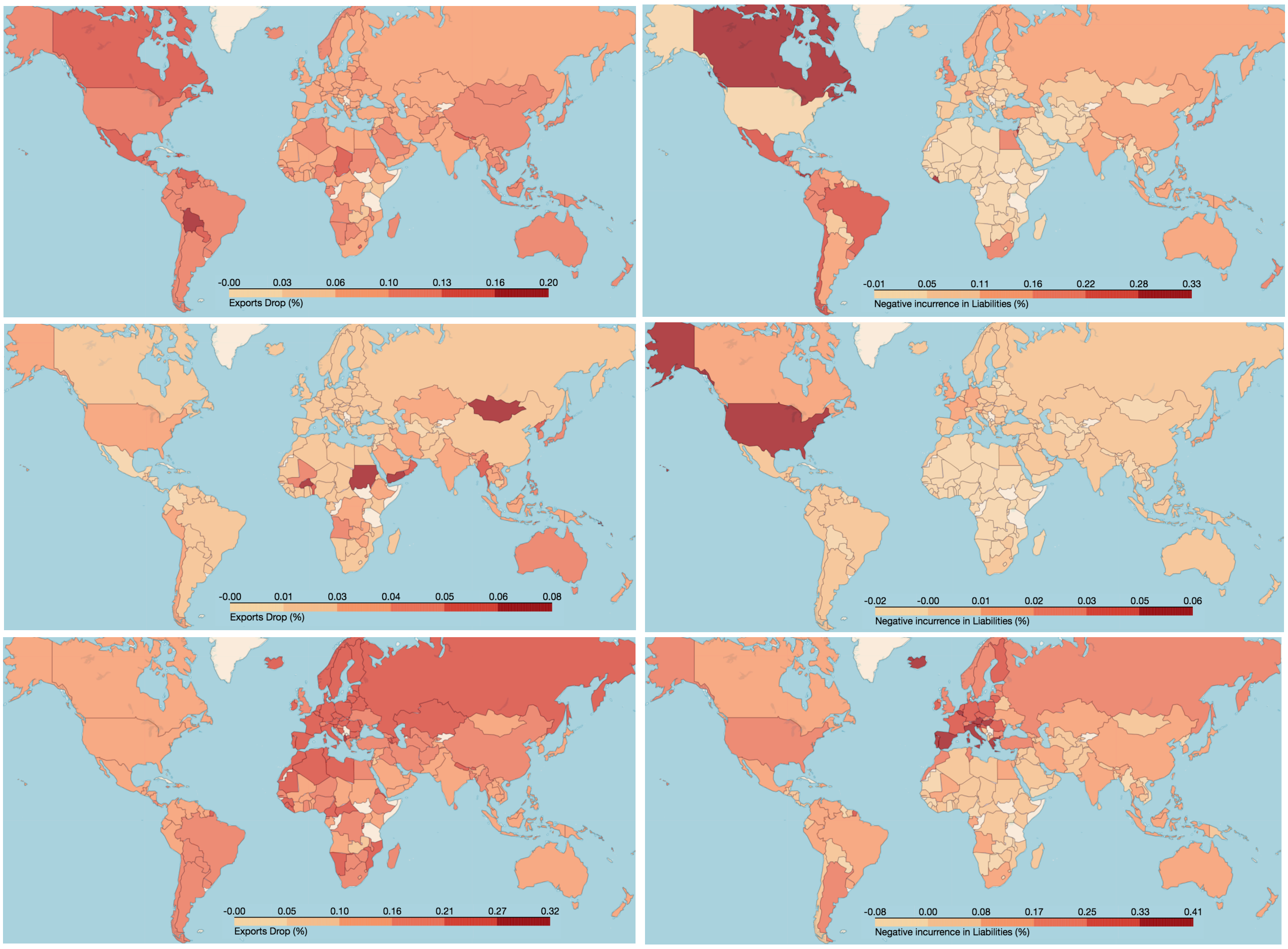

Figure 6: Vulnerability of each country with respect to a shock originated in the United States (first row), China (second row), or in countries belonging to the EZ (third row), characterized by $\alpha = -0.4$ and $\beta = -0.1$. Colors indicate the average of exports, $VaR[\Delta X_i]$ (left plots), and of incurrence in liabilities, $VaR[\Delta L_i]$ (right plots).

VI. QUANTIFYING SYSTEMIC IMPACT OF EPICENTER COUNTRIES

Here we show the systemic impact as a function of the magnitude of the initial shock (Fig. 7), and the deviations from the linear relation (Fig. 8). These Figures correspond to Fig. 3 of the main text, with different values of α and β.

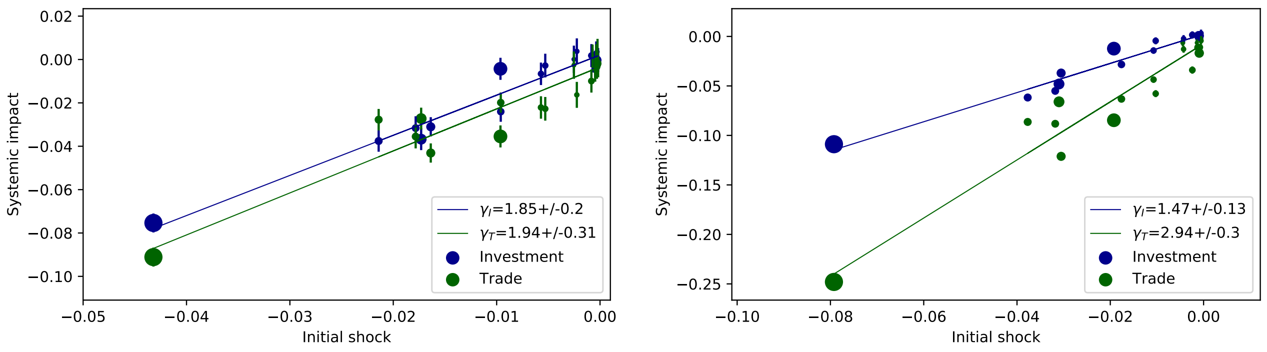

Figure 7: Systemic impact on global trade $\mathcal{S}_i^T$ and investment $\mathcal{S}_i^I$, as a function of the magnitude of the initial shock $\mathcal{I}_i/(W_I + W_T)$. The initial shock is characterized by $\alpha = -0.1, \beta = -0.3$ (left), or $\alpha = -0.3, \beta = -0.5$ (right). Countries belonging to the G_{20} group are shown. Error bars represent the standard error of the mean for $\mathcal{S}_i$. Regression coefficients γ_ℓ are plotted with 95% CI. Size of dots is proportional to countries' GDP.

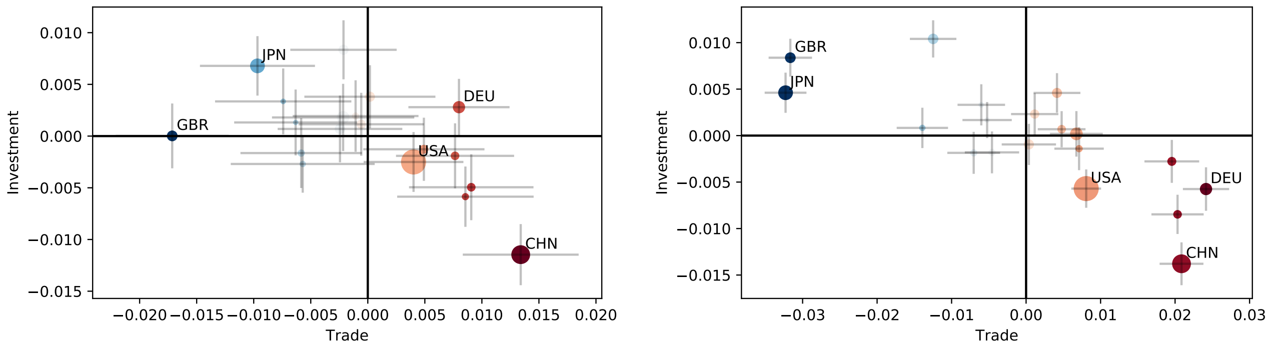

Figure 8: Trade (x-axis) versus financial (y-axis) deviations, as obtained by Figure 7. The initial shock is characterized by $\alpha = -0.1, \beta = -0.3$ (left), or $\alpha = -0.3, \beta = -0.5$ (right). Countries belonging to the G_{20} group are shown. Error bars represent the standard error of the mean for the systemic impact. Size of dots is proportional to countries' GDP.

VII. NETWORK MULTIPLIERS PREDICT SYSTEMIC IMPACT

Here we show the systemic impact as a function of the an initial shock originated only in the investment (Fig. 9) or trade (Fig. 10) layer. We also show the comparison between actual and predicted systemic impacts, obtained by means of the network multipliers presented in the main text. These Figures correspond to Figs. 4 and 5 of the main text, with different values of α and β.

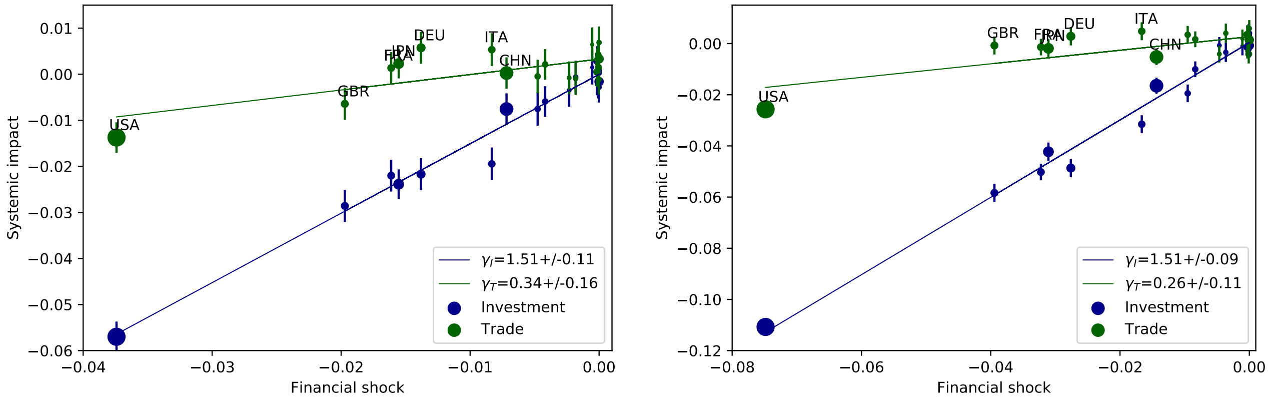

Figure 9: Systemic impact on global trade $\mathcal{S}_i^T$ and investment $\mathcal{S}_i^I$, as a function of the an initial shock $\mathcal{I}_i^\ell/W_\ell$ originated only in the investment layer, for $\beta = -0.2$ (left) or $\beta = -0.4$ (left), for countries belonging to the G_{20} group. Error bars represent the standard error of the mean for $\mathcal{S}_i$. Regression coefficients $\gamma_{\ell' \to \ell}$ are plotted with 95% CI. Size of dots is proportional to countries' GDP.

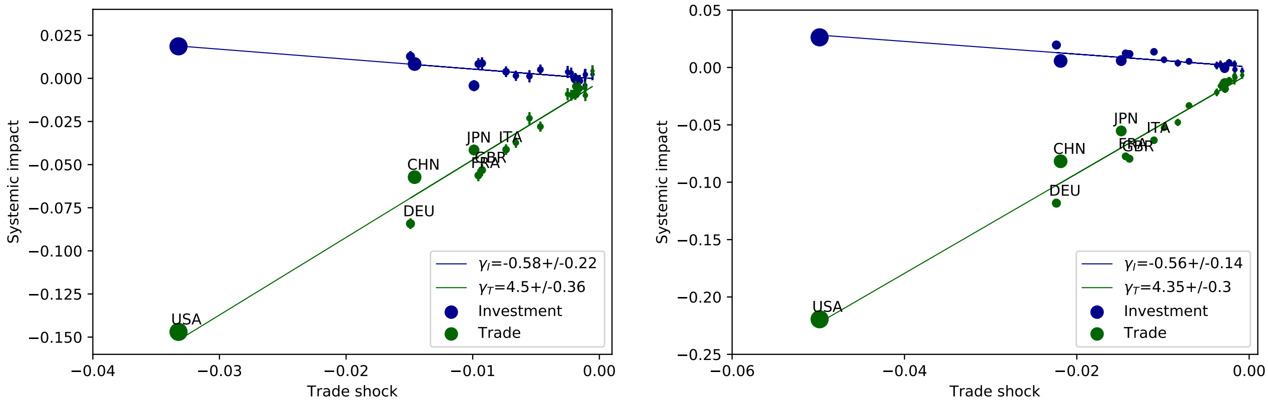

Figure 10: Systemic impact on global trade $\mathcal{S}_i^T$ and investment $\mathcal{S}_i^I$, as a function of the an initial shock $\mathcal{I}_i^\ell/W_\ell$ originated only in the trade layer, for $\alpha = -0.2$ (left) or $\alpha = -0.3$ (right), for countries belonging to the G_{20} group. Error bars represent the standard error of the mean for $\mathcal{S}_i$. Regression coefficients $\gamma_{\ell' \to \ell}$ are plotted with 95% CI. Size of dots is proportional to countries' GDP.

[1] Guillermo García-Pérez, Marián Boguñá, Antoine Allard, and M. Ángeles Serrano, "The hidden hyperbolic geometry of international trade: World trade atlas 1870–2013," Scientific Reports **6**, 33441 EP – (2016).

[2] Gabriel Zucman, "The missing wealth of nations: Are europe and the u.s. net debtors or net creditors?*," The Quarterly Journal of Economics **128**, 1321–1364 (2013).

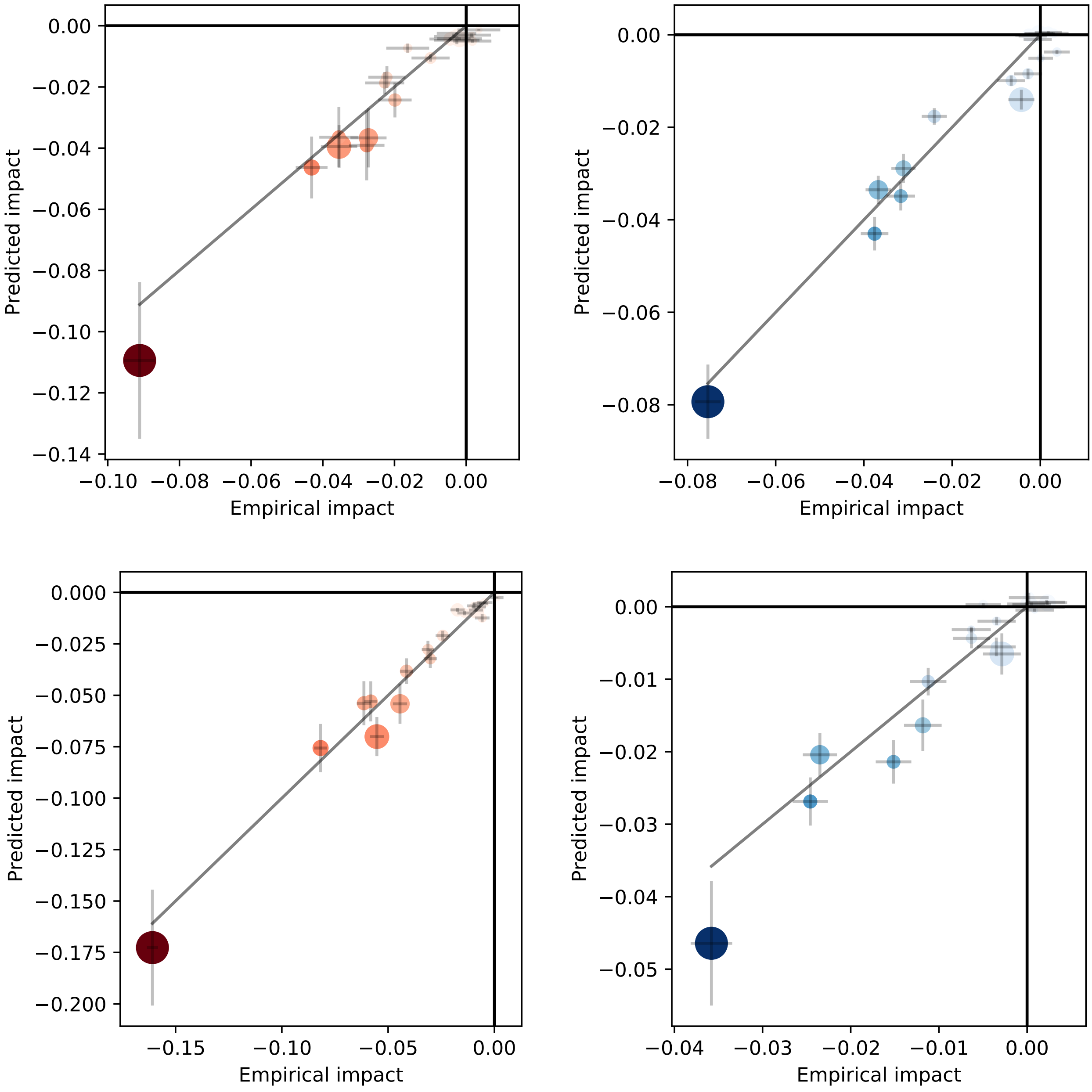

Figure 11: Expected versus actual systemic impact on trade (left) and investment (right) of each country i belonging to the G_{20} group, originated by an initial shock with $\alpha = -0.1, \beta = -0.3$ (top), or with $\alpha = -0.2, \beta = -0.2$ (bottom). The size of dots is proportional to their GDP, color proportional to $\mathcal{S}_i^\ell$ (red for $\ell = T$, blue for $\ell = I$). Uncertainties are represented by grey crosses.
